# HOPX regulates bone marrow-derived mesenchymal stromal cell fate determination via suppression of adipogenic gene pathways

**DOI:** 10.1038/s41598-020-68261-2

**Published:** 2020-07-09

**Authors:** Chee Ho Hng, Esther Camp, Peter Anderson, James Breen, Andrew Zannettino, Stan Gronthos

**Affiliations:** 1 Mesenchymal Stem Cell Laboratory, South Australian Health and Medical Research Institute, North Terrace, Level 5, Adelaide, SA 5001 Australia; 2 Mesenchymal Stem Cell Laboratory, Adelaide Medical School, Faculty of Health and Medical Sciences, University of Adelaide, Adelaide, SA Australia; 3 Adelaide Craniofacial Unit, Women and Children Hospital, North Adelaide, SA Australia; 4 Robinson Research Institute, University of Adelaide, Adelaide, SA Australia; 5 Bioinformatics Hub, University of Adelaide, Adelaide, SA Australia; 6 Myeloma Research Laboratory, Adelaide Medical School, Faculty of Health and Medical Sciences, University of Adelaide, Adelaide, SA Australia

**Keywords:** Mesenchymal stem cells, Cell biology, Stem cells

## Abstract

Previous studies of global binding patterns identified the epigenetic factor, EZH2, as a regulator of the homeodomain-only protein homeobox (HOPX) gene expression during bone marrow stromal cell (BMSC) differentiation, suggesting a potential role for HOPX in regulating BMSC lineage specification. In the present study, we confirmed that EZH2 direct binds to the *HOPX* promoter region, during normal growth and osteogenic differentiation but not under adipogenic inductive conditions. *HOPX* gene knockdown and overexpression studies demonstrated that HOPX is a promoter of BMSC proliferation and an inhibitor of adipogenesis. However, functional studies failed to observe any affect by HOPX on BMSC osteogenic differentiation. RNA-seq analysis of *HOPX* overexpressing BMSC during adipogenesis, found HOPX function to be acting through suppression of adipogenic pathways associated genes such as ADIPOQ, FABP4, PLIN1 and PLIN4. These findings suggest that HOPX gene target pathways are critical factors in the regulation of fat metabolism.

## Introduction

Human bone marrow derived mesenchymal stromal cells (BMSC) are a stem/progenitor cell population with some capacity for self-renew and the ability to differentiate into multiple lineages including osteoblasts, adipocytes, chondrocytes and smooth muscle cells^1–3^. BMSC are quiescent cells in vivo but can readily proliferate following ex vivo expansion to form clonogenic adherent colonies with variable growth and differentiation potentials^2,4^. However, the precise molecular mechanisms that maintain BMSC growth, self-renewal and cell fate determination are yet to be determined.

The homeodomain-only protein homeobox gene, *HOPX*, encodes the smallest known member of the homeodomain-containing protein family^5,6^. Unlike other typical homeobox proteins that bind to DNA and regulate their expression, HOPX does not bind directly to DNA, but rather it binds to different protein partners, acting as a co-factor to regulate molecular mechanisms by recruiting transcription factors to gene promoter regions^5–7^. Previous studies have demonstrated an association between Enhancer of zeste homolog 2 (EZH2) and HOPX in BMSC, where *EZH2* was reported to be a repressor of *HOPX* expression^8,9^. EZH2 is a histone methyltransferase that trimethylates the histone 3 lysine 27 (H3K27me3), which then leads to chromatin condensation and gene repression^10^. In BMSC, EZH2 inhibits osteogenesis and cellular senescence, while allowing adipogenesis to occur^9,11^, implicating a possible role for HOPX during BMSC growth and differentiation.

*HOPX* expression has been identified in many tissues, and is a critical protein in cardiac development^5,6^. Various studies with conflicting data reported that HOPX is a critical factor in maintaining the balance between cellular proliferation and differentiation by promoting or inhibiting different molecular pathways^5,6,12,13^. Currently, no known function of HOPX has been identified during BMSC growth or differentiation. Using loss-of-function and gain-of-function studies, we demonstrated that HOPX is a promoter of proliferation and inhibitor of adipogenesis in human BMSC.

## Materials and methods

All methods were performed in accordance with The University of Adelaide and Australian Health & Medical Research Council guidelines and regulations. Human bone marrow samples were isolated from normal healthy adult donors with informed consent, in accordance to the guidelines and regulations of the Royal Adelaide Hospital Human Ethics Committee (protocol No. 940911a).

### Isolation and culture of BMSC

Human BMSC were isolated from iliac crest derived bone marrow mononuclear cells from normal adults (18–30 years of age) following STRO-1 positive selection using FACS. The BMSC were cultured in normal growth media (αMEM supplemented with 10% fetal calf serum (FCS), 2 mM l-glutamine, and 100 μM l-ascorbate-2-phosphate) at 37 °C with 5% CO_2_ as previously described^2^.

### RNA isolations and cDNA synthesis

Total RNA from 2 × 10^5^ human BMSC cultures (day 7–14 of osteogenic or adipogenic induction) was extracted using Trizol (Invitrogen) in accordance with the manufacturer’s instructions. RNA (1 μg) was then used as a template for cDNA synthesis using Superscript IV Reverse Transcriptase (Invitrogen LifeTechnologies, Carlsbad, CA). The expression levels of transcripts were assessed by semi-quantitative real-time polymerase chain reaction (qPCR) amplification as previously described^14^. Primer sets used in this study:

*ADIPOQ* (Fwd: 5′-cctaagggagacatcggtga-3′; Rev: 5′-gtaaagcgaatgggcatgtt-3′),

*ADIPSIN* (Fwd: 5′-gacaccatcgaccacgac-3′; Rev: 5′-ccacgtcgcagagagttc-3′),

*AOC3* (Fwd: 5′-gtctttgtccccatggct-3′; Rev: 5′-cacttgttgctgtggttgct-3′),

*C/EBPα* (Fwd: 5′-gggcaaggccaagaagtc-3′; Rev: 5′-ttgtcactggtcagctccag-3′),

*CNN1* (Fwd: 5′-aggctccgtgaagaagatca-3′; Rev: 5′-ccacgttcaccttgtttcct-3′),

*FABP4* (Fwd: 5′-tactgggccaggaatttgac-3′; Rev: 5′-gtggaagtgacgcctttcat-3′),

*G0S2* (Fwd: 5′-ggaagatggtgaagctgtacg-3′; Rev: 5′-cttgcttctggagagcctgt-3′),

*GPD1* (Fwd: 5′-aaacgccactggcatatctc-3′; Rev: 5′-tttggtgtctgcatcagctc-3′),

*HOPX *(Fwd: 5′-tcaacaaggtcgacaagcac-3′; Rev: 5′-gtgacggatctgcactctga-3′),

*OPN* (Fwd: 5′-gcagacctgacatccagtacc-3′; Rev: 5′-gatggccttgtatgcaccattc-3′),

*PLIN1* (Fwd: 5′-ctctcgatacaccgtgcaga-3′; Rev: 5′-tggtcctcatgatcctcctc- 3′),

*PLIN4* (Fwd: 5′-ccttcggaaaagatggtgtc-3′; Rev: 5′-taagtgcagaccgagtggtg-3′),

*RUNX2* (Fwd: 5′-gtggacgaggcaagagtttca-3′; Rev: 5′-catcaagcttctgtctgtgcc-3′),

*β-ACTIN* (Fwd: 5′-gatcattgctcctcctgagc-3′; Rev: 5′-gtcatagtccgcctagaagcat-3′).

### Chromatin immunoprecipitation

Human BMSC (1 × 10^6^) were cultured under normal growth, osteogenic or adipogenic inductive conditions. Chromatin immunoprecipitation (ChIP) was performed using the Magna ChIP kit (Millipore Corporation, Billerica, MA, https://www.merckmillipore.com.au) according to the manufacturer’s instructions. An anti-rabbit EZH2 antibody (49-1043, Life Technologies, Mulgrave, VIC, Australia) and anti-rabbit IgG control antibodies (ab171870, Abcam, Melbourne, Australia) were used for the immunoprecipitation. Levels of immunoselected genomic DNA was then assessed using PCR as previously described^9^. ChIP primer sets: *GAPDH* (Fwd: 5′-tgtcagtgcgttccagtctc-3′, Rev: 5′-aggaacaggagggaaaagga-3′), *p14TSS* (Fwd: 5′-ggagcgatgtgatccgttatc-3′, Rev: 5′-tgaaatcccaatcgtcttccac-3′), *HOPX* (S1) (Fwd: 5′-tgctcatctgttggaaaacg-3′, Rev: 5′-caactccccttcctccaaat-3′), *HOPX* (S2) (Fwd: 5′-tcccacagatgatctcacca-3′, Rev: 5′-tgcatgcagagtgtgacaga-3′), *HOPX* (S3) (Fwd: 5′-aagcccacaggtggaagttt-3′, Rev: 5′-gttccccgcaagacaagtta-3′). *EZH2* binding sites S1, S2 and S3 on *HOPX gene* are shown in Supplementary Fig. 1.

### Retroviral transduction

Full-length human coding sequence for *HOPX* (NCBI RefSeq: NM_001145459.1) was subcloned into the pRUF-IRES-GFP vector (Kind gift by Paul Moretti, University of South Australia, Australia). Retroviral transduction of *HOPX*/pRUF-IRES-GFP or empty vector control pRUF-IRES-GFP into human BMSC was performed as previously described^15^. Stably transduced BMSC expressing high levels of GFP were selected by FACS, using a BD FACSAria Fusion flow cytometer (https://www.bdbiosciences.com). Overexpression of *HOPX* was confirmed by qPCR analysis.

### siRNA knock-down transfections

Human BMSC were seeded at 10^4^ cells/cm^2^ and siRNA knockdown was performed on the following day. Sequence specific siRNAs against *HOPX* (ThermoFisher Scientific, https://www.thermofisher.com/) were used at 12 pMol to achieve a > 90% knockdown of transcript levels. The siRNA used in this study were: *HOPX* s39106 and s39107 and Silencer Select Negative Control #1 siRNA. The procedure was performed in accordance with manufacturer’s instructions with a 72 h incubation period before performing functional assays.

### BrdU proliferation assay

Proliferation assay (4–6 days) was performed in accordance with the manufacturer’s instructions using the Cell Proliferation ELIZA, BrdU kit (11647229001; Roche Diagnostics Corporation, Indianapolis, IN).

### In vitro* differentiation assays*

Human BMSC (10^4^ cells/cm^2^) were cultured in either normal growth conditions (αMEM supplemented with 10% FCS, 2 mM l-glutamine, and 100 μM l-ascorbate-2-phosphate); or osteogenic inductive conditions (αMEM supplemented with 5% FCS, 2 mM l-glutamine, 50 U/mL penicillin–streptomycin, 10 mM HEPES buffer, 1 mM sodium pyruvate, 0.1 mM dexamethasone, 100 μM l-ascorbate-2-phosphate and 2.6 mM KH_2_PO_4_); or adipogenic inductive conditions (αMEM supplemented with 10% FCS, 2 mM l-glutamine, 50 U/mL penicillin–streptomycin, 10 mM HEPES buffer, 1 mM sodium pyruvate, 120 mM indomethacin and 0.1 mM dexamethasone) for up to 3 weeks as previously described^15,16^. Mineralized bone matrix was assessed with Alizarin red (Sigma-Aldrich, Inc.) staining^15^. Extracellular calcium was measured as previously described^15^. Lipid formation was identified by Nile-red (Sigma-Aldrich, Inc.) staining as previously described^15^. Quantitation of lipid was assessed by Oil Red O staining (MP Biomedicals, Solon, OH), Nile-red fluorescence staining normalized to DAPI stained nuclei per field of view in triplicate wells as previously described^15^.

### RNA-sequencing

BMSC with either empty vector and *HOPX* overexpressing vector were cultured at 2.5 × 10^4^ cells in normal growth (Ctrl) or adipogenic (Adipo) inductive media for 2 weeks. RNA was isolated and purified using Trizol (Sigma-Aldrich Inc., Sydney, NSW, Australia) in accordance with manufacturer’s instructions. 1 μg of RNA was processed and sequenced by David Gunn Genomic Facility, SAHMRI, SA, Australia on the Illumina Nextseq 500 with a 75 cycle v2.5 High Output sequencing kit. Initial raw read processing was performed using an in-house pipeline developed at SAHMRI. Raw 75 bp single-end FASTQ reads were assessed for quality using FastQC^17^ and results aggregated using R/Bioconductor package *NgsReports*^18^. Reads were then trimmed for sequence adapters using *AdapterRemoval*^19^ and aligned to the human genome GRCh38/hg38 using the RNA-seq alignment algorithm *STAR*^20^. After alignment, mapped sequence reads were summarised to the GRCh38.p13 (NCBI: GCA_000001405.28) gene intervals using *FeatureCounts*^21^, and count table transferred to the R statistical programming environment for expression analysis. Effect of sequence duplicates were also investigated using the function *MarkDuplicates* from the Picard tools package (https://broadinstitute.github.io/picard).

### Differential gene expression and pathway analysis

Gene expression analyses were carried out in R using mostly Bioconductor packages *EdgeR*^22,23^ and *Limma*^24^. Gene counts were filtered for low expression counts by removing genes with less than 1 count per million (cpm) in more than two samples and then normalised by the method of trimmed mean of M-values^25^. Differential gene expression was carried out on log-CPM counts and precision weights available from the *Voom* function in *Limma*^26^, with linear modelling and empirical Bayes moderation. Annotation of results were carried out using Ensembl annotations (https://grch37.ensembl.org) available in *BiomaRt*^27^, and expression results displayed in heatmaps using the *Pheatmap* package^28^.

### Statistics

Generation of graphs and data analysis was performed using GraphPad Prism 7 (GraphPad Software, LA Jolla, CA, https://www.graphpad.com/). Statistical significance (*) of p < 0.05 between samples are shown based on Student’s t-test and One-way ANOVA as indicated.

## Results

### HOPX expression is directly repressed by EZH2

**Figure 1:**
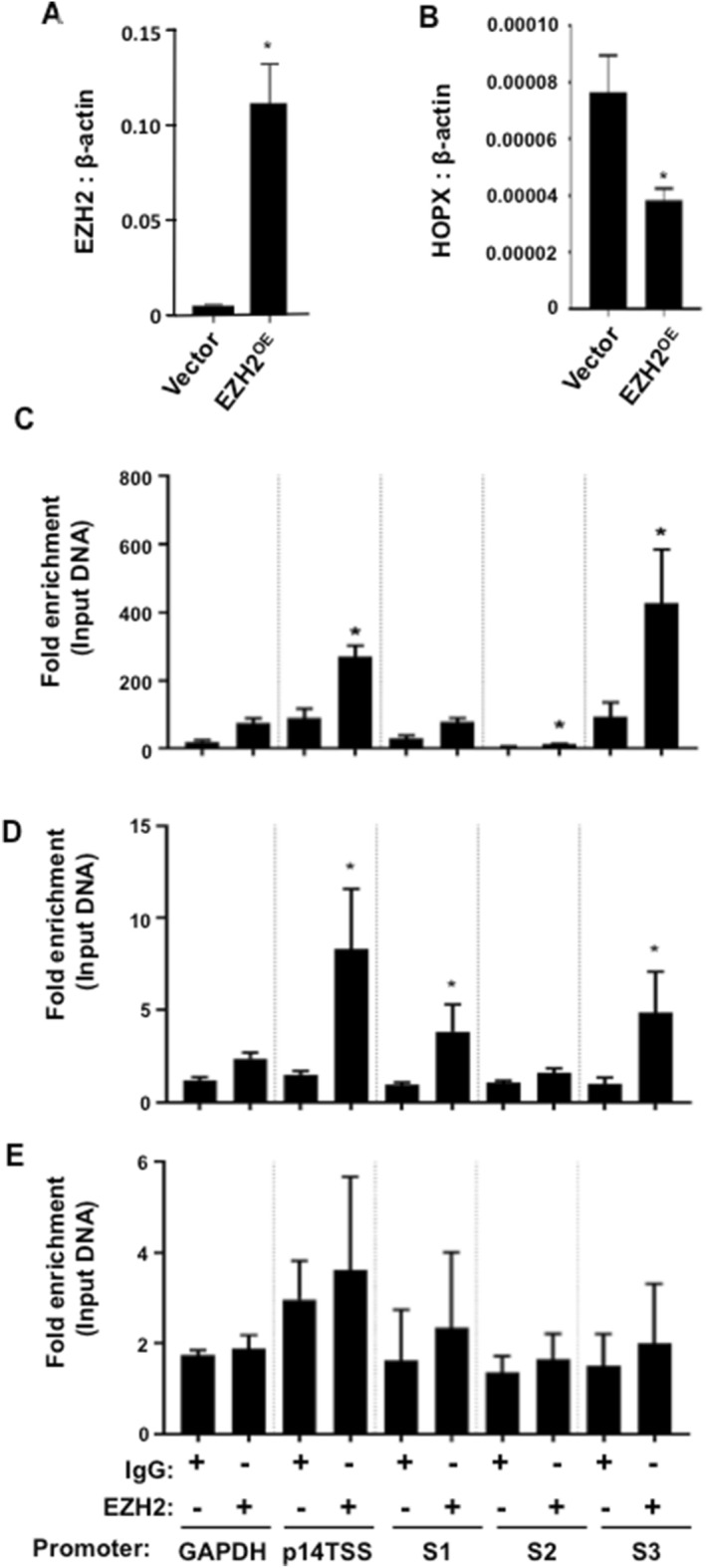
HOPX gene expression is upregulated during BMSC differentiation and is suppressed by EZH2. Stable *EZH2* overexpressing BMSC (EZH2^OE^) and Vector control BMSC were analysed for (**A**) *EZH2* and (**B**) *HOPX* gene expression levels using qPCR relative to *β-ACTIN*. Error bars represent mean ± S.E.M, n = 3 donors. (**C**–**E**) ChIP analysis of three putative *EZH2* binding sites located on the *HOPX* promoter regions (site 1 (S1), site 2 (S2) and site 3 (S3) see Supplementary Fig. 1) using control antibody (IgG) or EZH2 antibody (EZH2). Fold enrichment was calculated by measuring the levels of enriched genomic DNA compared to the input genomic DNA of BMSC cultured in (**C**) normal growth, (**D**) osteogenic or (**E**) adipogenic inductive conditions for 1 week by PCR. Graph represents mean ± S.E.M enriched genomic DNA of *GAPDH* (negative control), *p14TSS* (positive control) and S1, S2, S3 of *HOPX* promoter regions, n = 3 BMSC donors. Fold enrichment results for *HOPX*, S1, S2 and S3 were compared to *GAPDH* (negative control). Student’s t-test p < 0.05(*).

Previous studies of global ChIPseq analyses found that the H3K27 methyltransferase, EZH2, regulates *HOPX* expression during BMSC osteogenic differentiation^8^. Enforced expression of EZH2 in cultured human BMSC resulted in a decrease in *HOPX* gene expression levels (Fig. 1A,B). Manual ChIP analysis was used to assess the binding of EZH2 to putative DNA binding sites on *HOPX*, using genomic DNA isolated from cultured human BMSC. The data showed preferential binding of EZH2 to the S3 binding region of the *HOPX* promoter region in BMSC cultured under normal growth conditions and osteogenic inductive conditions (Fig. 1C,D). However, EZH2 enrichment on all *HOPX* binding sites (S1, S2 and S3) was greatly diminished when BMSC were cultured under adipogenic inductive conditions (Fig. 1E).

### HOPX is a promoter of BMSC proliferation

**Figure 2:**
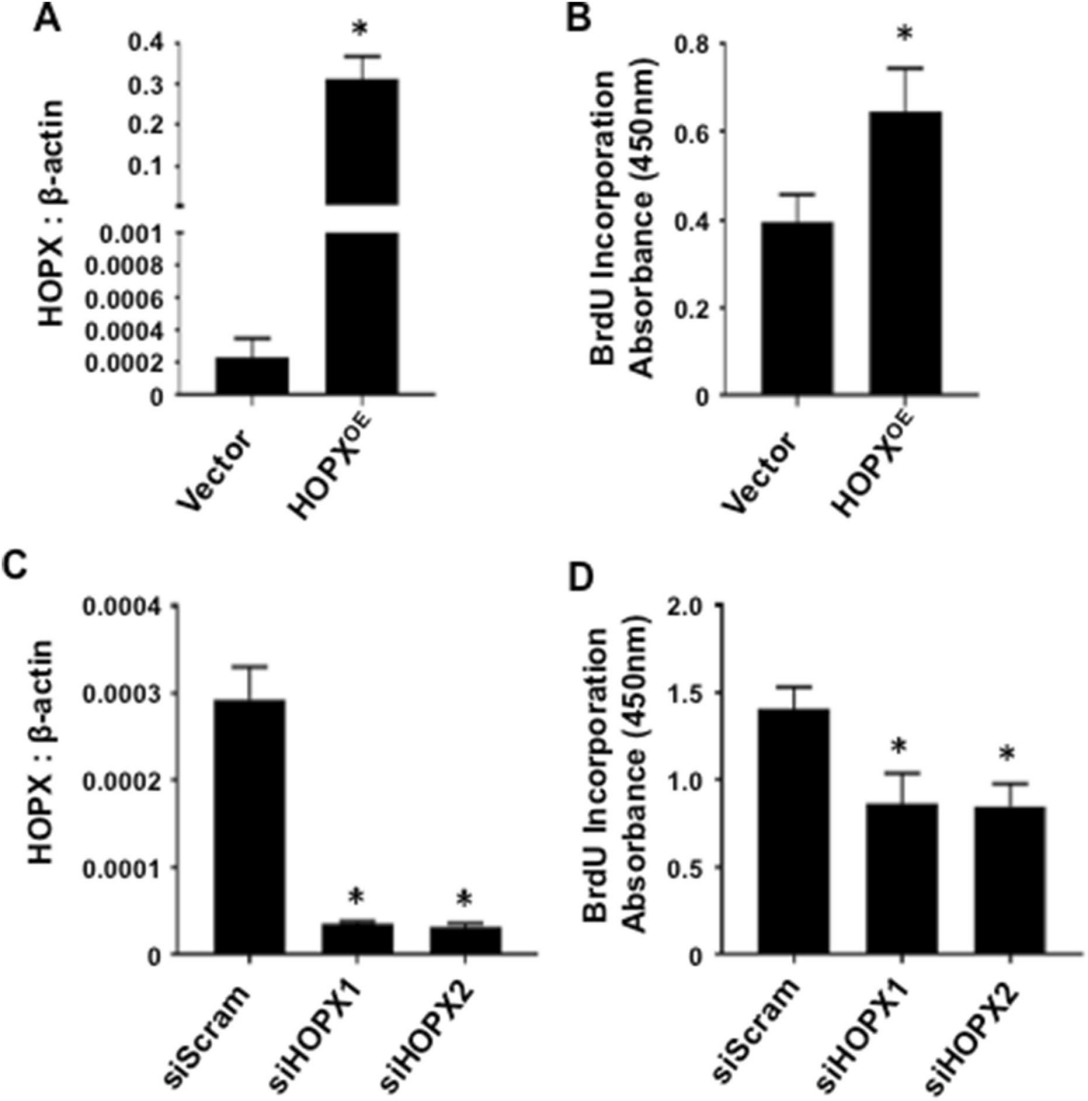
HOPX promotes BMSC proliferation. (**A**) First strand cDNA was prepared from total RNA harvested from *HOPX* overexpressing (HOPX^OE^) and Vector only BMSC, then analysed for *HOPX* gene expression levels using qPCR, relative to *β-ACTIN*, n = 5 donors. (**B**) HOPX^OE^ and Vector control BMSC were incubated for 4 days and analysed by BrdU assay, n = 4 donors. (**C**) cDNA was prepared from RNA harvested from BMSC treated with scramble siRNA (siScram) or siRNA targeting *HOPX* (siHOPX1 & siHOPX2). *HOPX* gene expression levels were analysed by qPCR relative to *β-ACTIN*, n = 4 donors. (**D**) siScram, siHOPX1 (n = 4 donors) and siHOPX2 (n = 3 donors) BMSC were incubated for 6 days and assessed for BrdU incorporation. Error bars represent mean ± S.E.M, Student’s t-test p < 0.05(*).

In order to determine if HOPX regulates BMSC proliferation, *HOPX* was overexpressed in BMSC using retroviral transduction (Fig. 2A). Cell proliferation was assessed by BrdU incorporation under normal growth conditions. The data showed a significant increase in the proliferation rates of BMSC following enforced expression of *HOPX* (Fig. 2B). To further confirm that HOPX regulates BMSC proliferation, *HOPX* expression was knocked down using two independent siRNA molecules targeting *HOPX* transcripts (Fig. 2C). Knock down of *HOPX* in BMSC resulted in a significant decreased in proliferation rates (Fig. 2D). These data suggest that HOPX is a positive regulator of BMSC proliferation.

### HOPX is an inhibitor of BMSC adipogenesis

**Figure 3:**
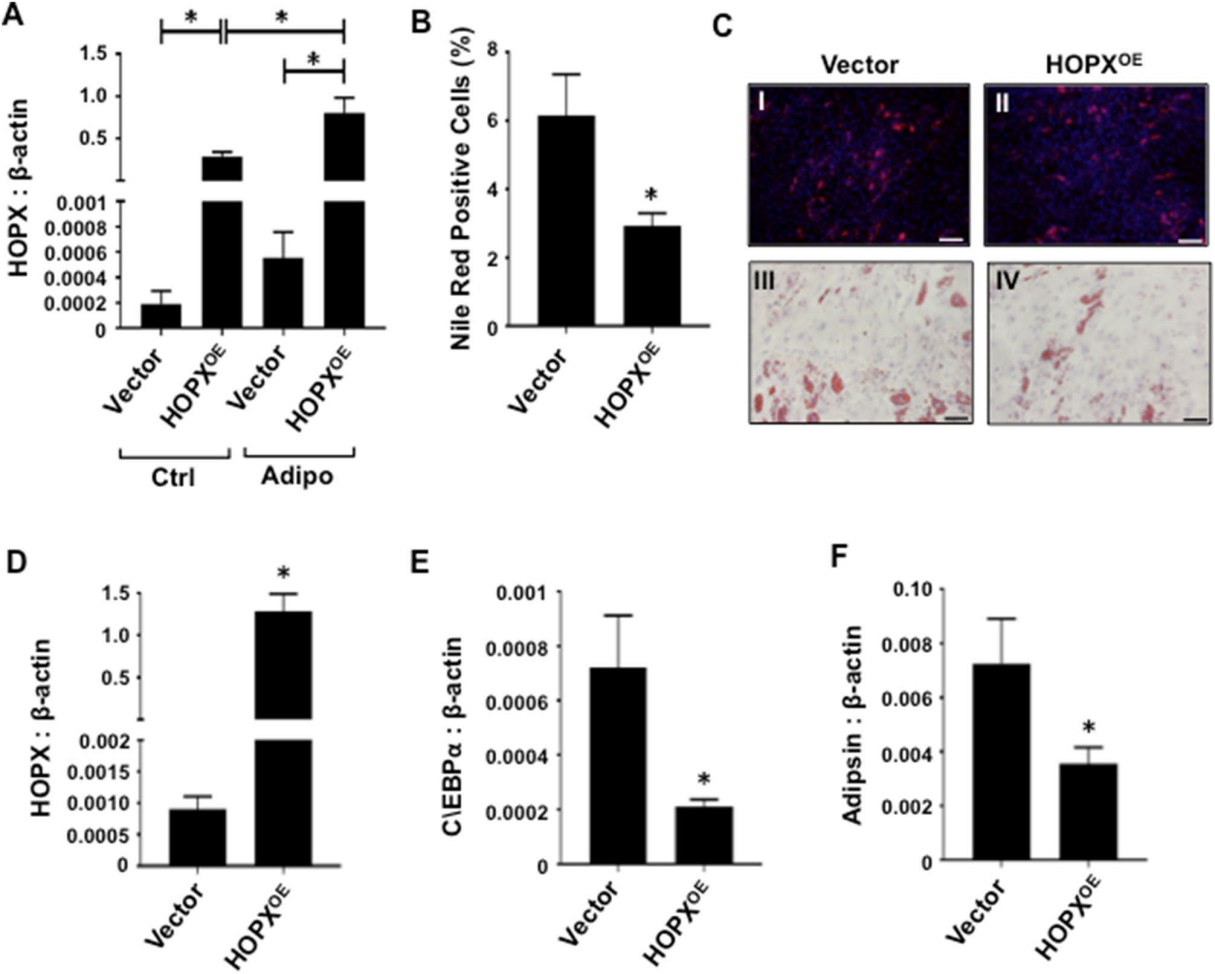
Enforced expression of HOPX inhibits BMSC adipogenesis. (**A**) *HOPX* overexpressing (HOPX^OE^) and Vector only BMSC were cultured in either control (Ctrl) or adipogenic (Adipo) conditions for 3 weeks and *HOPX* expression levels were determined relative to *β-actin* using qPCR, n = 6 donors. Error bars represent mean ± S.E.M, One-way ANOVA p < 0.05(*). (**B**) Lipid-containing HOPX^OE^ and Vector only BMSC stained with Nile-red and DAPI were quantified, n = 3 donors. (**C**) Representative images of lipid-containing (I) Vector control BMSC and (II) HOPX^OE^ BMSC stained with Nile-red and DAPI. (**C**) Representative images of lipid-containing (III) Vector control BMSC and (IV) HOPX^OE^ BMSC stained with Oil Red O. Total RNA was harvested at 7–14 days (n = 6 donors) post induction from HOPX^OE^ and Vector BMSC. Gene expression levels were measured by qPCR for (**D**) *HOPX*, (**E**) *C/EBPα* and (**F**) *ADIPSIN* relative to *β-ACTIN*. Error bars represent mean ± S.E.M, Student’s t-test p < 0.05(*). Scale bar (20 μm).

We next explored the role of HOPX during human BMSC differentiation. Functional studies were carried out using retroviral transduced *HOPX* overexpressing constructs or empty vector infected BMSC, cultured in control or adipogenic inductive media (Fig. 3A). Overexpression of *HOPX* resulted in decreased Nile-red-positive lipid producing adipocytes compared with empty vector control cells (Fig. 3B,C). Quantitative analysis of *HOPX* overexpressing BMSC lines showed a significant reduction of lipid-producing adipocytes compared with vector control BMSC (Fig. 3C). Expression levels of adipogenic gene transcripts were assessed using qPCR, following adipogenic induction. The data demonstrated that *HOPX* overexpressing BMSC (Fig. 3D) exhibited decreased levels of *C/EBPα* (Fig. 3E) and *ADIPSIN* (Fig. 3F) expression levels when compared to vector only BMSC, under adipogenic inductive conditions.

**Figure 4:**
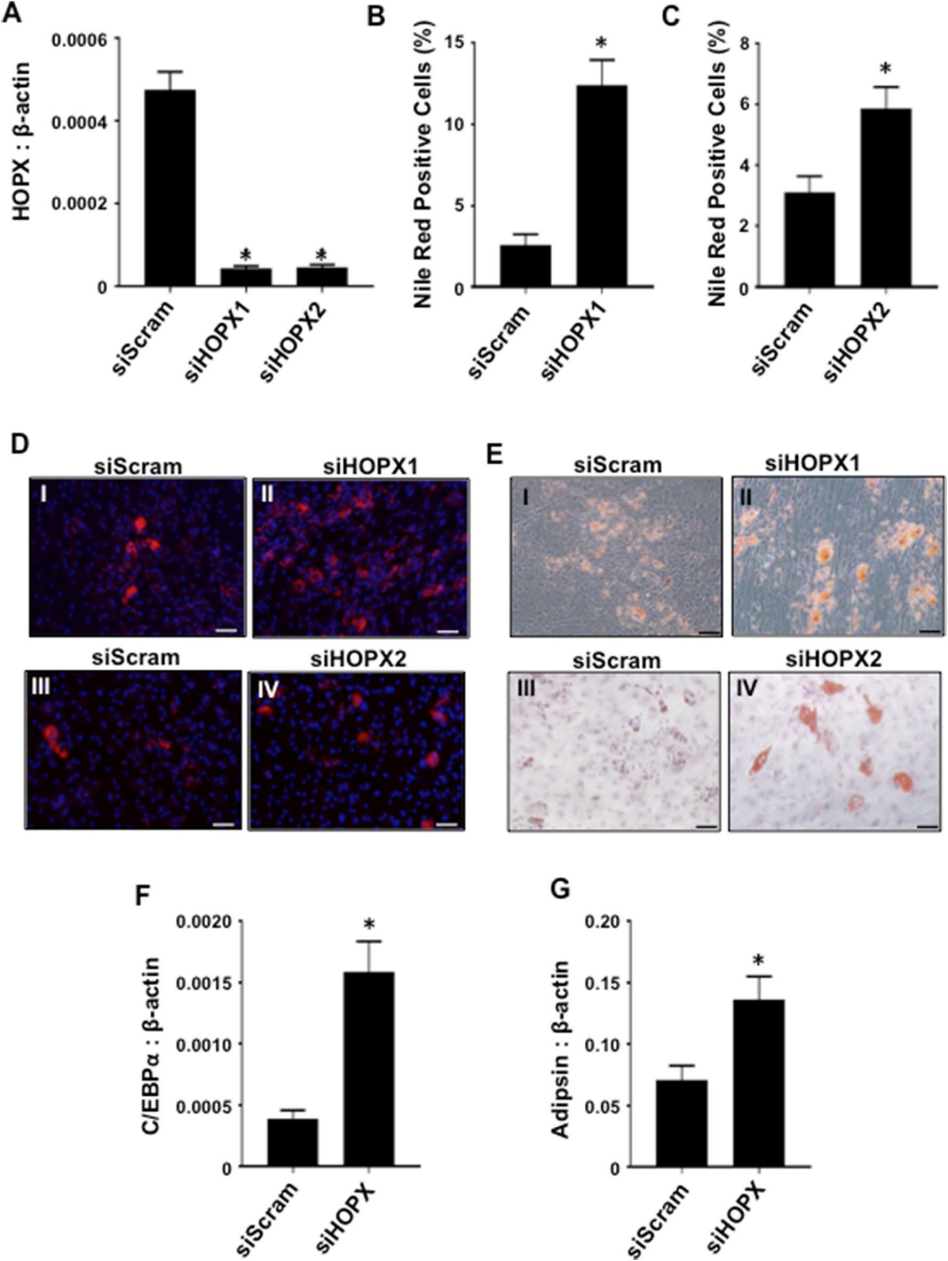
Knockdown of HOPX expression promotes BMSC adipogenesis. (**A**) siScram, siHOPX1 and siHOPX2 BMSC cultured in adipogenic (Adipo) inductive conditions for 3 weeks and *HOPX* expression levels determined relative to *β-ACTIN* using qPCR, n = 8 donors. (**B**) Lipid-containing cells treated with siScram or siHOPX1 stained with Nile-red and DAPI, then quantified, n = 3 donors. (**C**) Lipid-containing cells treated with siScram or siHOPX2 were stained with Nile-red and DAPI, then quantified, n = 4 donors. (**D**) Representative images of lipid-containing (I, III) siScram, (II) siHOPX1 and (IV) siHOPX2 BMSC stained with Nile-red and DAPI. (**E**) Representative images of lipid-containing (I, III) siScram, (II) siHOPX1 and (IV) siHOPX2 BMSC stained with Oil Red O. Total RNA was harvested at 7–14 days post induction from BMSC treated with siScram or siHOPX, n = 4 donors. Gene expression levels were measured by qPCR for (**F**) *C/EBPα,* (**G**) *ADIPSIN* relative to *β-ACTIN*. Error bars represent mean ± S.E.M, Student’s t-test p < 0.05(*). Scale bar (20 μm).

To verify these findings, siRNA-mediated knockdown using two independent siRNA targeting *HOPX* transcripts in BMSC was performed (Fig. 4A). The data showed a dramatic increase in Nile-red-positive lipid-producing adipocytes following adipogenic induction, compared with BMSC treated with control scramble siRNA (Fig. 4B–E). Furthermore, siRNA knockdown of *HOPX* resulted in an increase in *C/EBPα* (Fig. 4F) and *ADIPSIN* (Fig. 4G) transcript levels compared with scramble siRNA-treated cells following adipogenic induction. Overall, these data demonstrate that HOPX is a repressor of adipogenesis.

**Figure 5:**
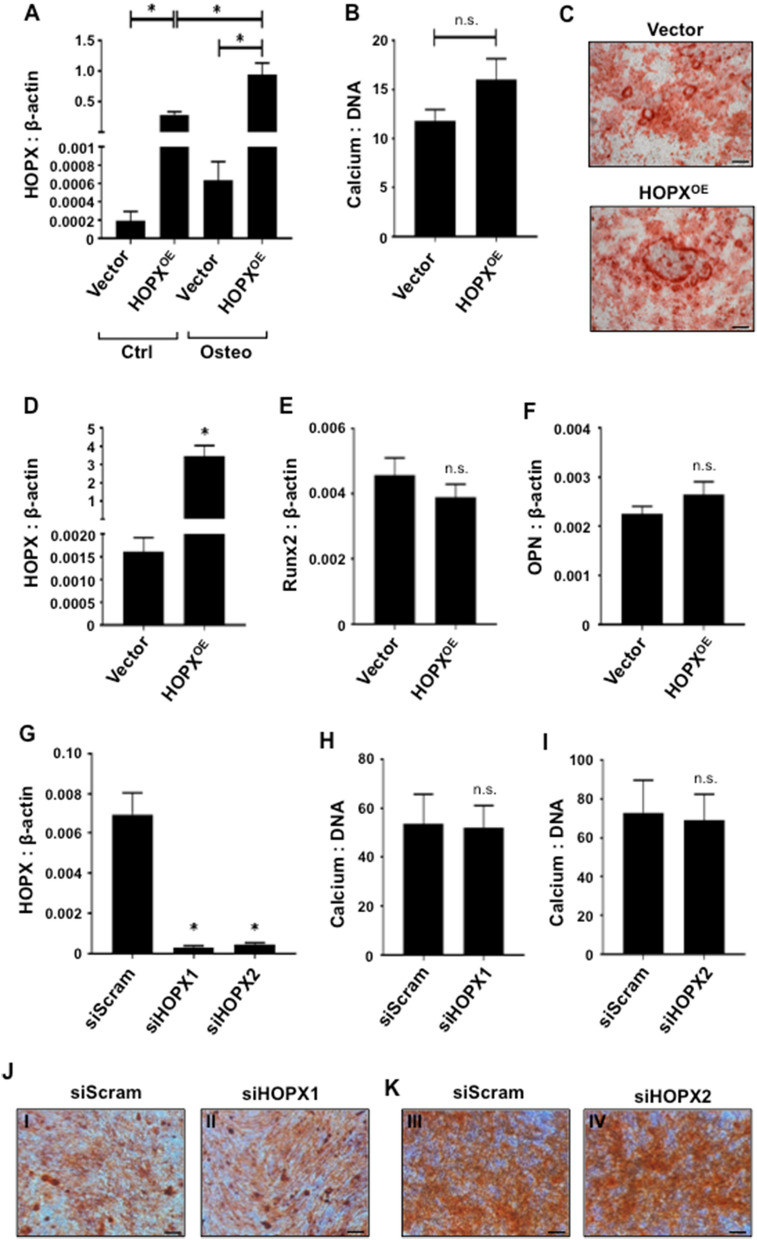
HOPX does not affect BMSC osteogenic differentiation. (**A**) *HOPX* overexpressing (HOPX^OE^) and vector only (Vector) BMSC were cultured in either control (Ctrl) or osteogenic inductive (Osteo) conditions and *HOPX* expression levels were determined relative to *β-ACTIN* using qPCR, n = 6 donors. Error bars represent mean ± S.E.M, One-way ANOVA p < 0.05(*). (**B**) Extracellular calcium levels were quantitated and normalized to total DNA content per well, n = 3 donors. (**C**) Vector control and HOPX^OE^ BMSC stained with Alizarin red. Total RNA was harvested at 7–14 days post induction (n = 6 donors) from Vector and HOPX^OE^ BMSC. Gene expression levels were measured by qPCR for (**D**) *HOPX*, (**E**) *RUNX2*, (**F**) *OPN* relative to *β-ACTIN*. (**G**) siScram, siHOPX1 and siHOPX2 BMSC were incubated in control (Ctrl) or osteogenic inductive (Osteo) conditions for 3 weeks, and *HOPX* expression levels were determined relative to *β-ACTIN* using qPCR, n = 8 donors. Extracellular calcium levels were quantitated in siScram, (**H**) siHOPX1 and (**I**) siHOPX2 BMSC and normalized to total DNA content per well, n = 4 donors. (**J**, **K**) (I, III) siScram, (II) siHOPX1 and (IV) siHOPX2 BMSC were stained with Alizarin red. Representative of one donor is shown. Error bars represent mean ± S.E.M, Student’s t-test p < 0.05 (*), n.s. represents non-significant. Scale bar (20 μm).

To identify the function of HOPX in BMSC osteogenic differentiation, *HOPX* overexpressing BMSC or empty vector infected BMSC were cultured under control or osteogenic inductive media (Fig. 5A). Assessments of extracellular calcium levels found no difference between *HOPX* overexpressing BMSC and vector control BMSC (Fig. 5B). Similarly, mineralized deposits were stained with Alizarin Red after 3 weeks under osteogenic growth conditions with no observable differences (Fig. 5C). In accord with these findings, *HOPX* overexpressing BMSC (Fig. 5D) showed no significant difference in the transcript levels of the osteogenic master regulator, *RUNX2* (Fig. 5E) and the mature bone marker, *OSTEOPONTIN (OPN)* (Fig. 5F), compared to the vector control cells. Confirmatory studies employing siRNA-mediated knockdown of *HOPX* in BMSC (Fig. 5G) found no significant differences in the levels of Alizarin positive mineral and extracellular calcium levels compared with scramble siRNA-treated BMSC (Fig. 5H–K). Overall, these findings demonstrate that HOPX has no direct effect on the osteogenic capacity of BMSC.

### *HOPX inhibits BMSC adipogenic differentiation *via* suppression of adipogenic associated genes*

**Figure 6:**
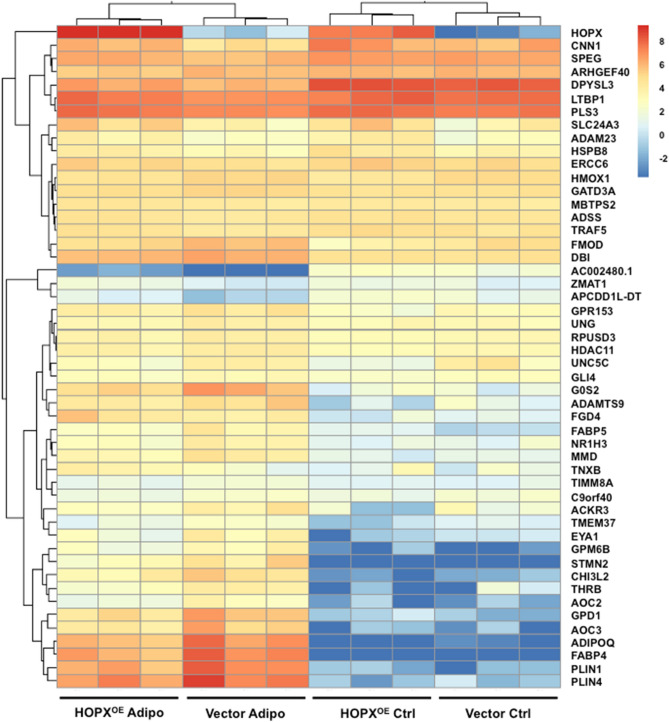
Potential mechanisms of HOPX regulation of BMSC adipogenesis. *HOPX* overexpressing (HOPX^OE^) and Vector only BMSC were cultured in either control (Ctrl) or adipogenic inductive (Adipo) conditions for 2 weeks. Total RNA was collected and assessed by RNA-seq analysis, n = 3 donors per condition. The heat map depicts the top 50 differentially expressed genes (DE) selected based on fold expression as shown.

**Figure 7:**
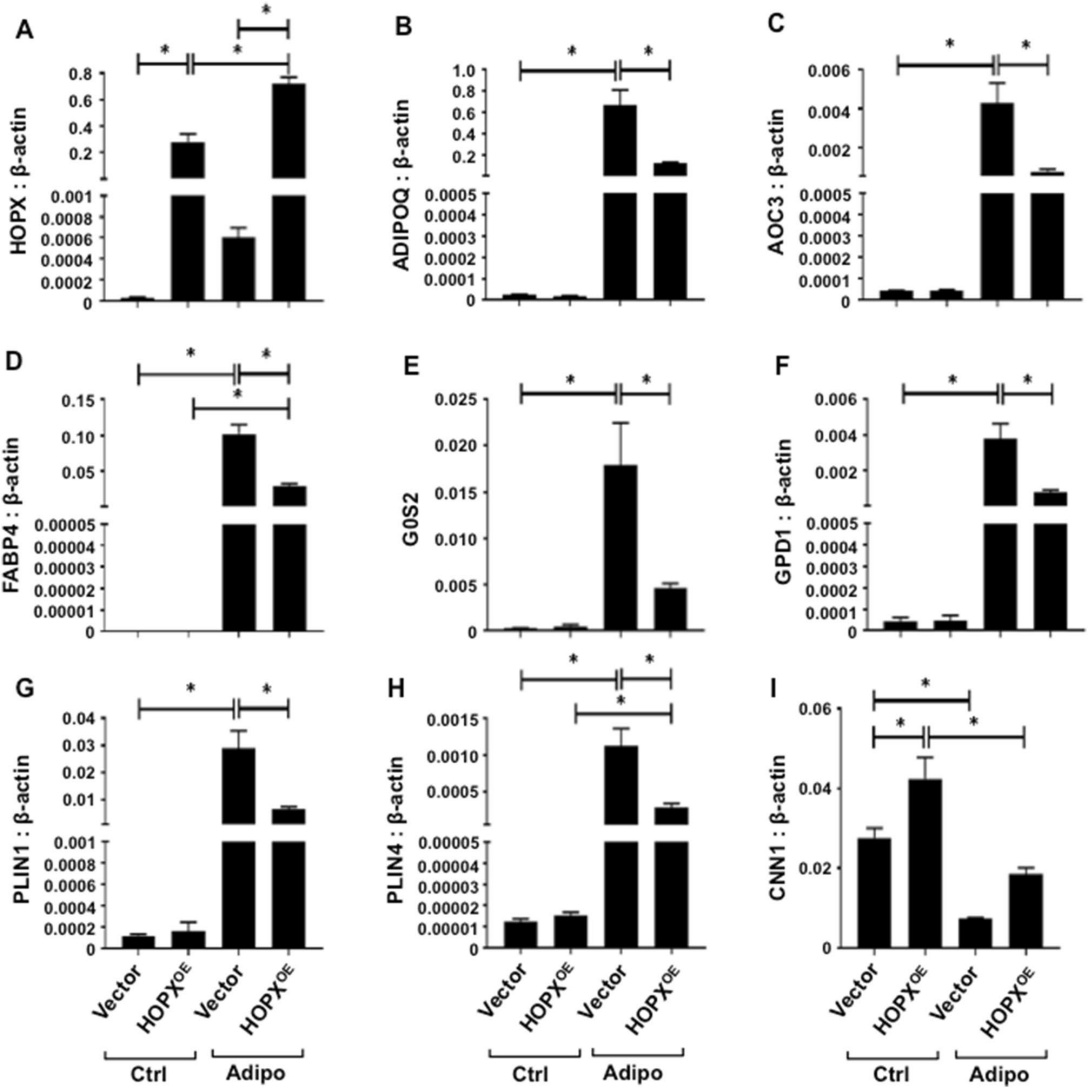
Confirmation of RNA-seq data. *HOPX* overexpressing (HOPX^OE^) and vector only (Vector) BMSC cultured under normal growth (Ctrl) or adipogenic inductive conditions (Adipo) then assessed by qPCR to measure transcript levels for (**A**) *HOPX*, (**B**) *ADIPOQ*, (**C**) *AOC3*, (**D**) *FABP4*, (**E**) *G0S2*, (**F**) *GPD1*, (**G**) *PLIN1*, (**H**) *PLIN4*, (**I**) *CNN1* relative to *β-ACTIN*. Error bars represent mean ± S.E.M, One-way ANOVA p < 0.05(*), n = 2 donors.

We next explored potential mechanisms of HOPX action during BMSC adipogenic differentiation. Total RNA was collected from *HOPX* overexpressing and vector control BMSC cultured for 2 weeks under adipogenic inductive conditions, then processed for RNA-sequencing to identify novel HOPX-regulated genes during BMSC adipogenic commitment [79]. Due to the variable gene expression patterns between different individuals (n = 3 donors), the P-value significance was excluded as a criteria to select for differentially expressed genes (DE). Therefore, the top 50 DE (Fig. 6) were selected based on the fold change (a log fold change (logFC) ≥ |1| or ≥ |− 1|). To validate the RNA-sequencing results, confirmatory qPCR was performed on a number of genes that appeared to change expression in *HOPX* overexpressing BMSC under adipogenic conditions. HOPX transcripts were found to be elevated in *HOPX* overexpressing BMSC compared to vector control BMSC, which were relatively higher during adipogenesis compared to normal growth conditions for the respective population. From the transcriptional expression heat map (Fig. 6), we observed a number of genes that were upregulated during adipogenesis but suppressed in HOPX overexpressing cells. Table 1 indicates the functional role of these genes following Gene Ontology (GO) enrichment analysis, with 188 genes involved in EMT, 185 genes in adipogenesis and 127 genes in fatty acid metabolism. The differential gene expression levels of representative upregulated genes, *HOPX*, *ADIPOQ*, *AOC3*, *FABP4*, *G0S2*, *GPD1*, *PLIN1* and *PLIN4* were confirmed by qPCR (Fig. 7A–H). Other genes were found to be downregulated during adipogenesis and promoted by HOPX expression such as *CNN1* (Fig. 7I). The RNA-sequencing analysis provides insight into putative targets of HOPX during BMSC adipogenesis.

**Table 1:** Gene ontology annotations of differentially expressed genes from RNA-seq analysis of HOPX overexpressing and Vector only BMSC cultured under normal growth or adipogenic conditions.

Gene ontology	No. genes	Direction	p value	FDR
Epithelial mesenchymal transition	188	Down	6.01E−09	3.00E−07
Adipogenesis	185	Up	3.55E−07	8.88E−06
Hypoxia	161	Down	6.56E−05	0.001069
Bile acid metabolism	67	Up	8.55E−05	0.001069
Fatty acid metabolism	127	Up	1.96E−04	0.001964
Xenobiotic_metabolism	131	Up	4.19E−04	0.003494
Angiogenesis	23	Down	7.99E−04	0.00571
Glycolysis	157	Down	0.001059	0.006621

## Discussion

Our studies suggest that HOPX mediates postnatal BMSC proliferation and lineage determination. Protein structural studies have demonstrated that HOPX is unable to bind to DNA, suggesting that HOPX functions through protein–protein interaction with partner proteins. A number of HOPX partner proteins have been identified, including Hdac1, Hdac2, MTA 1/2/3, MBD3 and Rbbp4/7^29^. *HOPX* has been shown to be a key factor in cardiac development, where it regulates cell proliferation and differentiation at different stages during murine cardiac development^5,6^. The present study found that human BMSC express low levels of *HOPX* during normal growth yet expression is dramatically increased during osteogenesis and adipogenesis in agreement with previous observations^8^.

Studies of homozygous mutations of the *Hopx* gene (loss-of-function mutations) in mouse showed partial penetrant embryonic lethality due to heart deformation during embryo development. However, those that survived display no gross deformities. *Hopx* heterozygous mutated mice are viable and comparable to wild type mice^5,6^. This suggests that Hopx is important for cardiac development. On the other hand, the incomplete penetrance of *HOPX* mutation indicates that there are other compensatory mechanisms that rescue part of the phenotype. However, no bone or fat-associated phenotypes have been reported in *Hopx* knockout studies. Functional studies using siRNA-mediated knockdown of *HOPX* did not affect BMSC osteogenesis but did alter the cellular proliferation and adipogenic potential of the cells. Our studies showed that siRNA-mediated *HOPX* knockdown in human BMSC decreased proliferation and increased adipogenic potential of these cells. This was demonstrated by an increase of lipid formation and increased expression of early adipogenic marker *C/EBPα* and mature marker *ADIPSIN*, when compared to the siRNA scramble controls. Conversely, these results were confirmed by enforced expression of *HOPX* in BMSC using retroviral transduction. *HOPX* overexpressing BMSC demonstrated decreased lipid formation and decreased expression of adipogenic associated markers. Our data suggest that HOPX is a novel molecular inhibitor of BMSC adipogenesis, which may have implications in the regulation of fat metabolism. Furthermore, *HOPX* overexpression or knockdown studies, failed to demonstrate any effects on the osteogenic potential of BMSC. We predict that HOPX acts by inhibiting adipogenesis via suppression of C/EBPα by potentially binding to adipogenic suppressor proteins that act on its promoter region as a complex. Given that EZH2 inhibits BMSC osteogenic differentiation but allows adipogenesis to proceed^11,15^, implicates HOPX as a potential counter balance to regulate BMSC adipogenesis.

HOPX is known to repress transcription by direct interaction with co-repressors such as HDAC2, which consequently inactivate GATA6/Wnt7 pathway important in development and differentiation^7^. However, conflicting data in the literature demonstrate the duo-functions of HOPX in promoting and inhibiting proliferation and differentiation at different developmental stages, suggesting the importance of HOPX in maintaining the balance between growth and differentiation in various tissues based on in vitro and in vivo systems^5,6^. Our data suggests that in humans, HOPX is likely to play a role in fat metabolism.

In bone marrow, the differentiation of MSC into osteoblasts and adipocytes is competitively balanced. The commitment of BMSC to the adipogenic lineage may result in increased adipocyte formation and decreased osteoblast numbers as observed in age-related bone loss^30^. Numerous in vitro experiments performed on BMSC have revealed various factors that promote adipocyte formation inhibit osteogenesis, and conversely, many factors that promote osteoblast formation inhibit adipogenesis^31,32^. This occurs through the interaction between different signaling pathways such as Wnt, Bmp, TGF-β, Notch, mTOR^33–37^. Previous findings implicate the Bmp/Wnt signaling pathways in regulating HOPX family members^29^. Inhibition of HOPX in mouse and zebrafish results in disruption of cardiac development and lethality. HOPX is found to be expressed in cardiomyoblasts, which interacts physically with activated Smad4 and functions to coordinate local Bmp signals to inhibit Wnt pathway, promoting cardiomyogenesis^29^. However, little is known about the biological function of HOPX in BMSC during postnatal skeletal development and homeostasis.

In order to identify novel HOPX target genes during BMSC adipogenesis, RNA-seq analysis was performed on *HOPX* overexpressing and vector control BMSC cultured under normal growth or adipogenic inductive condition for 2 weeks. Differentially expressed genes were identified between normal growth and adipogenic inductive conditions. Survey of the literature identified 188 genes involved in EMT, 185 genes in adipogenesis and 127 genes in fatty acid metabolism. To identify possible signaling or molecular pathways involved in HOPX signaling, gene ontology (GO) enrichment analysis was performed. A heatmap was constructed according to the fold change of gene expression between *HOPX* overexpressing and vector control BMSC cultured under either normal growth or adipogenic conditions. Many of the top 50 differentially expressed genes were found to be associated with adipogenesis such as *ADIPOQ*, *FABP4*, *PLIN1* and *PLIN4*, which generally showed a negative correlation with *HOPX* expression.

ADIPOQ is a cytokine secreted in various tissues including BMSC^38^. Adiponectin signals through its cell surface receptors adipoR1 (adiponectin receptor 1) and adipoR2 (adiponectin receptor 2) and can act in either endocrine, paracrine or autocrine pathway^39,40^. Upon ligand binding, distinct signaling pathways are initiated across tissues including PPARα, mTOR, AMPK^41–43^. On the other hand, the downstream signaling of adipoR1 can stimulate oxidative phosphorylation, which subsequently increases cell differentiation via suppression of the Wnt inhibitor, sclerostin^44,45^. Therefore, suppression of ADIPOQ by HOPX leads to termination of various pro-adipogenic signaling pathways and results in decreased adipogenic potential of BMSC.

Interestingly, *CNN1* gene expression was increased in *HOPX* overexpressing BMSC compared to vector control BMSC, suggesting that *CNN1* is positively regulated by HOPX. CNN1 is an actin binding protein (ABP) that regulates the dynamics of actin cytoskeleton by direct/indirect participating in the assembly/disassembly of actin filament, which in turn regulates the cell contraction and movement^46^. CNN1 has been shown to play a role in bone homeostasis, where high expression of CNN1 leads to delayed bone formation and decreased bone mass^47,48^. CNN1 is known to interact directly with activated or inactivated Smad1/5/8 protein and inhibit Bmp2-Smad1/5/8 signaling^49^. Although the function of CNN1 in the regulation of fat metabolism is unknown, it is involved in the Bmp/Smad pathway, which is a critical pathway in the crosstalk between BMSC osteogenesis and adipogenesis. However, more studies are needed to determine the effects of HOPX on the ‘stemness’ state of BMSC, and whether *HOPX* is dysregulated during skeletal aging and bone disease in vivo, which are often associated with increased marrow adipogenesis at the expense of bone formation.

Collectively, our findings suggest that HOPX promotes human BMSC proliferation and inhibits adipogenesis, and this is the first ever finding showing the importance of the HOPX in human BMSC self-renewal and cell fate determination as a possible counter balance to EZH2 function (Supplementary Fig. 2), which normally represses HOPX gene expression in BMSC under normal growth conditions. HOPX appears to act by inhibiting BMSC adipogenesis via suppression of C/EBPα and potentially through co-factors binding to adipogenic suppressor proteins. Our future studies will employ co-immunoprecipitation and ChIPseq analyses to identify putative binding partners/co-factors of HOPX, the genome wide binding sites of HOPX protein complexes and the role of putative HOPX targets in human BMSC growth and lineage determination. This study lays the foundation for further research into the role of the homeobox family members in BMSC biology and fat metabolism.

## Supplementary information

Supplementary Legends.

Supplementary Figure 1.

Supplementary Figure 2.

## Data Availability

RNAseq data sets were downloaded to: https://www.ebi.ac.uk/ena/about/data-release-mechanism. Study accession number: PRJEB33928; Study unique name is: ena-STUDY-SAHMRI-08-08-2019-06:51:48:954-335.
