# Supplementary material for: HOPX regulates bone marrow-derived mesenchymal stromal cell fate determination via suppression of adipogenic gene pathways
Source: Sci Rep. 2020 Jul 9;10:11345. doi: 10.1038/s41598-020-68261-2 (PMC7347885; doi:10.1038/s41598-020-68261-2)
Supplement: Supplementary file 1 — Supplementary Legends. [file 41598_2020_68261_MOESM1_ESM.docx]

**Supplementary Figure 1. EZH2 binding sites on HOPX gene.** *In silico* analysis showing putative binding sites of EZH2 on the *HOPX* gene showing the exact positions of the Ezh2 binding sites (S1, S2 and S3) on HOPX gene (NC_000004.12 Reference GRCh38.p13 Primary Assembly) as suggested by the GTRD ChIP-seq database. Binding site co-ordinates on *HOPX* gene: S1 (-23361bp to -23273bp from TSS); S2 (-24313 to -24286 from TSS); S3 (+1165bp to +1263bp from TSS).

**Supplementary Figure 2.** **Model for HOPX regulation of BMSC adipogenesis.** EZH2 negatively regulates HOPX, which in turn mediates suppression of adipogenic genes such as *C/EBPα*, *ADIPOQ*, *FABP4*, *PLIN1* and *PLIN4*.
