# Supplementary figures and images for: HOPX regulates bone marrow-derived mesenchymal stromal cell fate determination via suppression of adipogenic gene pathways
Source: Sci Rep. 2020 Jul 9;10:11345. doi: 10.1038/s41598-020-68261-2 (PMC7347885; doi:10.1038/s41598-020-68261-2)

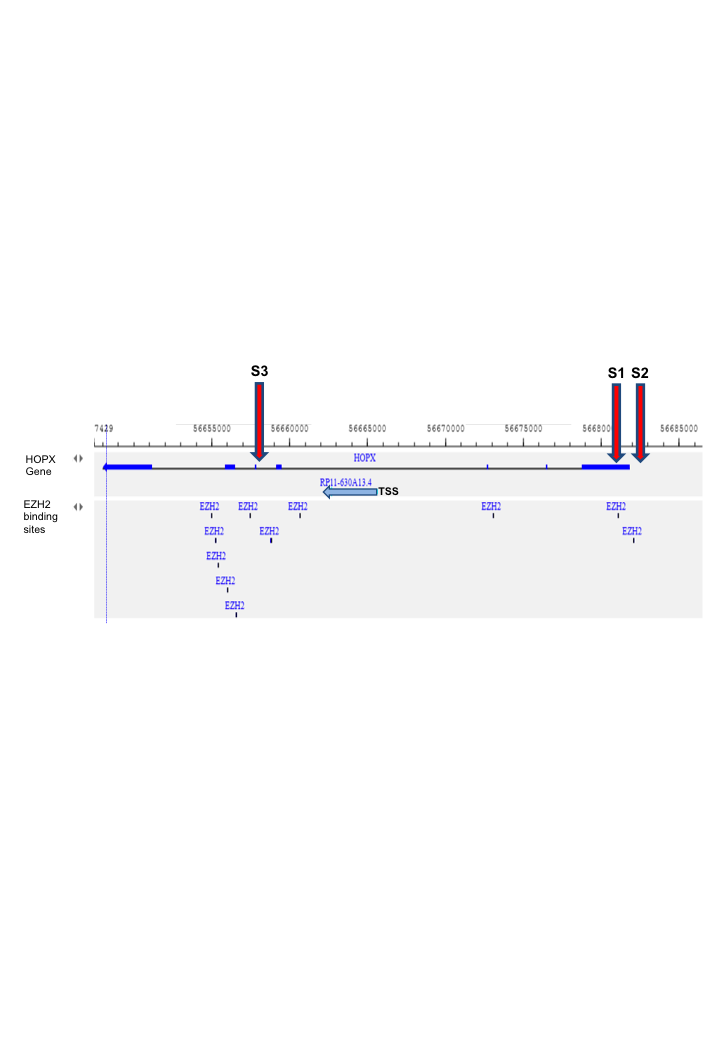

Supplement: Supplementary file 2 — Supplementary Figure 1. [file 41598_2020_68261_MOESM2_ESM.tiff]

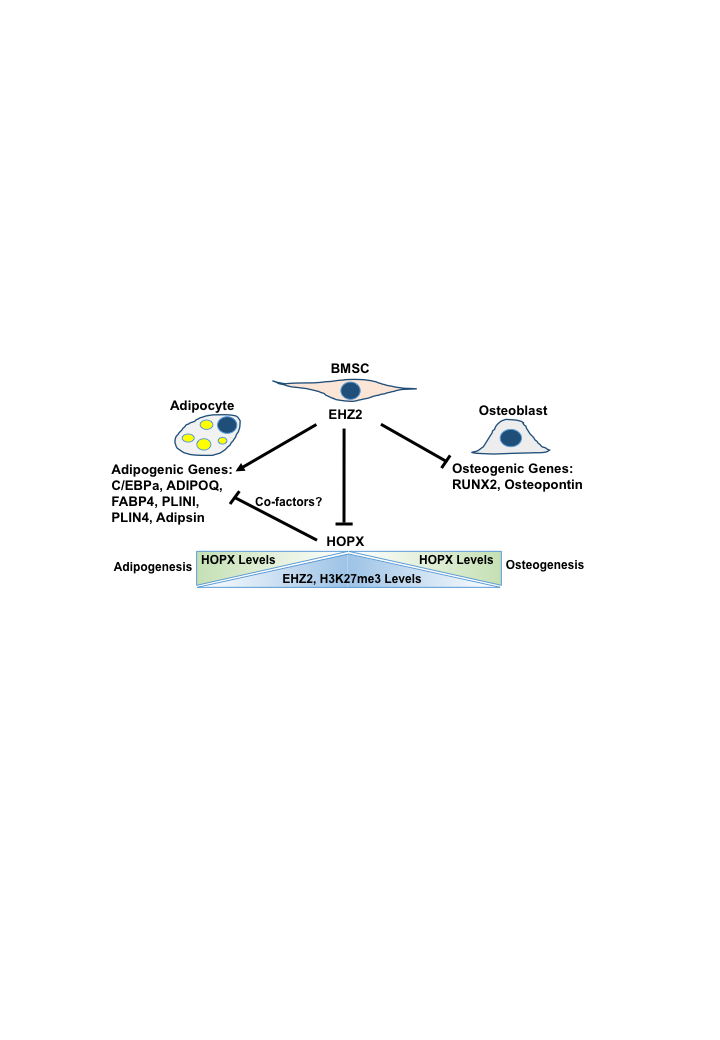

Supplement: Supplementary file 3 — Supplementary Figure 2. [file 41598_2020_68261_MOESM3_ESM.tiff]
